# Species Distribution Models Reveal Varying Degrees of Refugia From the Invasive Asian Needle Ant for Native Ants Versus Ant‐Plant Seed Dispersal Mutualisms

**DOI:** 10.1002/ece3.70750

**Published:** 2025-01-16

**Authors:** Drew Kanes, Daniel Malagon, Ben Camper, Anna Hewitt, Simon Dunn, Eva Purcell, Sharon Bewick

**Affiliations:** ^1^ Clemson University USA

**Keywords:** *Aphaenogaster*, *Brachyponera chinensis*, disturbance, Great Smoky Mountains National Park, invasion, MaxEnt

## Abstract

The Asian Needle Ant, *Brachyponera chinensis* (Hymenoptera: Formicidae), has spread throughout a substantial portion of the southeastern United States where it has primarily been restricted to low elevations. We focused on the *B*. *chinensis* invasion in Great Smoky Mountains National Park (GSMNP). Records in and near the park represent some of the highest elevation locations of *B*. *chinensis* in North America. The goals of this study were to characterize the status of the *B*. *chinensis* invasion in GSMNP, to assess the role that disturbance and human visitation play in *B*. *chinensis* invasion within GSMNP, to identify the potential of *B*. *chinensis* to spread into higher elevations in the park and the southern Appalachians and to determine the impact that this might have on native species, including keystone seed‐dispersers within the *Aphaenogaster rudis* complex and their myrmecochorous plants. We surveyed GSMNP for *B*. *chinensis* at 45 sites, including sites that were burned during the 2016 Gatlinburg fire, sites with high human visitation, and undisturbed sites. We then built species distribution models (SDMs) for *B*. *chinensis* and some of the native species that *B*. *chinensis* is most likely to impact. This allowed us to assess the potential for high‐elevation refugia within the southern Appalachians. We did not find *B*. *chinensis* at any undisturbed sites in GSMNP. We did find *B*. *chinensis* at five high‐visitation sites. Field findings were consistent with our SDMs, which suggested that GSMNP's unique precipitation regimes may act as a barrier to invasion. Unfortunately, SDMs indicated moderate suitability for *B*. *chinensis* across a sizable proportion of the northern border of the park. This is a region where *B*. *chinensis* may have disproportionate impacts on myrmecochorous plant species. Thus, although southern Appalachian precipitation and temperature regimes may provide a refuge from *B*. *chinensis* at high elevations, this will not protect all species likely to be impacted by this invasive ant.

## Graphical abstract

Using extensive searching, habitat suitability models, and minimum convex polygons, our paper presents the *B*. *chinensis* invasion and establishment of supercolonies at multiple sites across GSMNP. Our suitability models suggest moderate *B*. *chinensis* invasion potential for a large portion of the park, and we suggest that human disturbance has facilitated the invasion of this species into the park thus far. Furthermore, we report via refugia models that native ants in the *Aphaenogaster* spp. complex may find refuge from *B*. *chinensis* at higher elevations in the park, but multiple native plant species display minimal probability of such refugia.

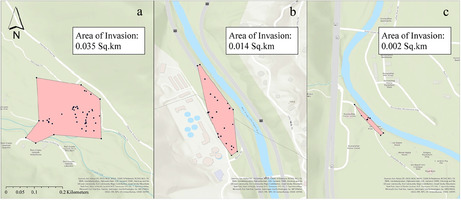

## 1 Introduction

One of the greatest threats faced by native biodiversity in the United States (U.S.) is biological invasion (Wilcove et al. 1998; Eiswerth and Johnson 2002; McClure et al. 2018). Since early European colonization, human‐facilitated dispersal (Mann 2005) has introduced over 50,000 invasive animal (Klima and Travis 2012; Dove et al. 2011; Hunter et al. 2015; Lewis et al. 2019; Robertson et al. 2020) and plant (Westbrooks 1998; Aurambout and Endress 2018; Lalk, Hartshorn, and Coyle 2021; Wickert et al. 2017) species into the American landscape. These invasive species have caused and continue to cause intense damage to native species through predation (Dove et al. 2011), competition (Bergstrom and Mensinger 2009), habitat destruction (Klima and Travis 2012), novel disease introduction (Scheele et al. 2019), and food web restructuring (Li et al. 2016; Bellard et al. 2014; Le Roux et al. 2019). Although invasive species are a concern everywhere, they are a particular threat in biodiversity hotspots and regions of high endemicity—areas that are both more likely to be invaded (Li et al. 2016; Bellard et al. 2014) and more likely to suffer species extinction as a result (Le Roux et al. 2019; Blackburn et al. 2004; Pyšek et al. 2017). One such region is the southern Appalachians (Adams et al. 2001). A world‐renowned biodiversity hotspot (Noss 2012; Hodkinson 2010; Milanovich et al. 2010; Irwin and Andrew 2000) across numerous taxa from salamanders to myrmecochores (plants that utilize ants as a significant means of seed dispersal) (Vander Wall and Moore 2016), the southern Appalachians derive much of their biodiversity from sensitive high‐elevation ecosystems (“sky islands”) (Caterino and Recuero 2024) that harbor large numbers of endemic and relict populations.

To date, the southern Appalachians have avoided many of the problematic invasive species that have colonized other regions (Iannone et al. 2016). This is likely because the old‐ and second‐growth forests that cover the southern Appalachians help to buffer against invasion (Warren et al. 2015; Oswalt et al. 2015). A paradigm of invasion biology is that invasive species preferentially thrive in open, human‐disturbed habitats lacking an intact canopy (Guénard and Dunn 2010; Martin, Canham, and Marks 2009; Richardson and Pyšek 2007). However, while generally less susceptible to invasion, the southern Appalachians have nevertheless been colonized by a variety of exotic pests, including some that have caused dramatic forest restructuring. Perhaps, the most notorious was chestnut blight ( *Cryphonectria parasitica* Murr. Barr) (McCormick and Platt 1980), a fungal pathogen that almost completely eradicated adult chestnuts, resulting in the conversion of oak‐chestnut forests to oak‐hickory forests (Opler 1977; Hough et al. 2024). This, in turn, led to the loss of native species that relied on chestnuts for food and shelter. A more recent invader, the hemlock wooly adelgid (*Adelges tsugae* Annand; Hemiptera: *Adelgidae*) (Ford et al. 2012), has also successfully colonized forests of the southern Appalachians. While the long‐term effects of the wooly adelgid invasion are still uncertain, dramatic changes are already being detected in communities ranging from stream invertebrates (Diesburg, Sullivan, and Manning 2019) to birds (Toenies et al. 2018). Thus, even though the majority of invasive species favor human‐disturbed habitats, invasive species that are capable of penetrating intact forests pose a substantial risk to Appalachian ecosystems. One such species is the invasive Asian needle ant (*Brachyponera chinensis* Emery; Hymenoptera: *Formicidae*)—a problematic pest that has recently reached the borders of the southern Appalachians and that appears to thrive in both urban environments and intact forests (Warren et al. 2015).

Ants comprise some of the most notorious invasive species. However, while ants are commonly transported outside of their native range by human activity (McGlynn 1999), most of these human‐introduced ant species either remain localized in a single location or else are strongly affiliated with human‐modified environments (so‐called “tramp ants”). Thus, as a whole, most introduced ant species have minimal ecological consequences (McGlynn 1999). Only a small number of ant species worldwide are truly invasive (Holway et al. 2002). However, ants that do become invasive can have devastating ecological impacts (McGlynn 1999; Majer 1985; Clark et al. 1982). Direct effects of invasive ants include displacement of native ant species, restructuring of ground‐dwelling invertebrate communities, and reduced recruitment of ground‐nesting birds and turtles. Invasive ants can also have myriad indirect effects on ecosystems. For example, the loss of native ants due to invasives has been associated with a reduction in food availability for mymechophagous predators and a reduction in seed dispersal of myrmecochorous plants. Certain invasive ant species may also decrease flower pollination by discouraging visitation by native pollinators. Fortunately, of the problematic invasive ant species currently found in North America, none has been able to penetrate the biodiverse, intact forests of the southern Appalachians. There is fear, however, that the southern Appalachians may pose less of an invasion barrier to *B*. *chinensis* as compared to other invasive ant species.

First identified in North America in 1932, *B*. *chinensis* has spread steadily throughout lower elevation forests in the southeastern U.S (Rodriguez‐Cabal et al. 2012; Campbell et al. 2019; Guénard, Wetterer, and Macgown 2018; Nelder et al. 2006; Yashiro et al. 2010). When present, *B*. *chinensis* rapidly dominates ant communities (Campbell et al. 2019; Warren, Bahn, and Bradford 2011) and reduces local ant diversity (Guénard and Dunn 2010; Guénard, Wetterer, and Macgown 2018). This is problematic because *B*. *chinensis* displaces native *Aphaenogaster* spp., disrupting seed‐dispersal mutualisms between *Aphaenogaster* ants and myrmecochorous plants (Rodriguez‐Cabal et al. 2012). In a comparative study of plots that were and were not invaded by *B*. *chinensis* , the presence of *B*. *chinensis* was associated with a 96% reduction in *A*. *rudis* , a 70% reduction in seed removal, and a 50% reduction in the population of a focal myrmecochore ( *Hexastylis arifolia* ). This is in keeping with current estimates that *Aphaenogaster* spp. are responsible for dispersing 61%–74% of mymecochorous seeds in eastern US forests (Ness, Morin, and Giladi 2009). Although mymecochores constitute only 9% of herbaceous species and 12% of herbaceous stems in eastern forests (Warren et al. 2021) overall, the southern Appalachians are a center of myrmecochore diversity. As a result, myrmecochores are both more diverse and more abundant throughout much of this region. In some Appalachian‐rich cove forests, for example, myrmecochores constitute > 60% of stems and > 40% of species richness (Handel, Fisch, and Schatz 1981; Beattie and Culver 1981; Ness, Morin, and Giladi 2009; Rico‐Gray and Oliveira 2008; Schultz 2014; Schultz et al. 2022). Thus, just as the invasive chestnut blight and hemlock wooly adelgid have restructured overstory trees of the southern Appalachians, *B*. *chinensis* has the potential to restructure understory plant communities in the forests of this region.

Although *B*. *chinensis* appears poised to spread into the southern Appalachians, it has thus far failed to do so to any considerable extent. While the failure of other invasive ant species to penetrate the southern Appalachians can be attributed to their inability to colonize intact forest, this is not the case for *B*. *chinensis* (Tschinkel and King 2017, 2013; Passera 2021). Thus, there has been speculation that the southern Appalachians may be protected, instead, by the limited ability of *B*. *chinensis* to invade elevations higher than ~600 m (Warren et al. 2020). Elevational records from *B*. *chinensis* in its native range, however, occur up to ~2450 m (Guénard, Wetterer, and Macgown 2018). Why *B*. *chinensis* occurs at different elevations in its native versus invasive range, and whether this means that the southern Appalachians are buffered from invasion remain open questions. Several hypotheses have been suggested for the restriction of *B*. *chinensis* to low elevations in eastern North America. One possible explanation is competition with other ant species that are present in high‐elevation communities in the southern Appalachians. This, however, is not consistent with ongoing displacement of the same ant species at lower elevations, nor is it consistent with aggression studies that have pitted *B*. *chinensis* against high‐elevation species like *Aphaenogaster* spp. (Warren et al. 2020) A second possible explanation is that *B*. *chinensis* cannot tolerate the colder temperature regimes at high elevations. This, however, is not consistent with the occurrence of *B*. *chinensis* at high elevations in its native range, where temperature regimes are similar. A third possible explanation is that there is an interaction between temperature and competition. Notably, *B*. *chinensis* has a higher critical thermal minimum (CT_min_) compared to co‐occurring *Aphaenogaster* spp (Warren et al. 2020), and this may give *Aphaenogaster* spp. a competitive advantage at higher elevations. A fourth possible explanation is that some other biotic or abiotic feature(s) unique to high elevations in the southern Appalachians may be preventing *B*. *chinensis* from invading. A fifth and most worrisome explanation is that the *B*. *chinensis* invasion may not have reached equilibrium (Lach 2021). In this case, current site occupancy reflects rate of colonization more than habitat suitability. Thus, colonized sites are those that are easily accessible (e.g., locations with high human visitation), while uncolonized sites are those that are less accessible (e.g., intact forests at higher elevations). However, because uncolonized sites are not intrinsically unsuitable, as the invasion proceeds and more and more dispersal barriers are overcome, these harder to access sites will also be invaded.

Recently, *B*. *chinensis* was found at mid‐elevation locations (~600 m) in the northern and southern foothills of Great Smoky Mountains National Park (GSMNP) and its surroundings (Paysen 2007). These locations represent the highest elevations where *B*. *chinensis* has been detected thus far in North America. Notably, *B*. *chinensis* occurrences at these locations have been at highly visited sites along the outskirts of the park (Paysen 2007). Given that *B*. *chinensis* is also proficient at colonizing disturbed and intact forest (Guénard, Wetterer, and Macgown 2018), it is unlikely that occurrences at high visitation sites reflect a requirement for disturbance. Rather, they likely reflect ease of dispersal to these locations. This makes our final explanation for the lack of *B*. *chinensis* in the southern Appalachians most likely: that the *B*. *chinensis* invasion is still ongoing and that high‐elevation southern Appalachian ecosystems will become invaded, given enough time to overcome dispersal barriers. To test the hypothesis that the *B*. *chinensis* invasion is still ongoing and that much of the southern Appalachians is at risk of *B*. *chinensis* colonization, we took a two‐pronged approach. First, we tested for evidence of ongoing spread of *B*. *chinensis* within GSMNP. Second, we built species distribution models (SDMs) to predict the potential for future spread of *B*. *chinensis* . If the *B*. *chinensis* invasion into the southern Appalachians is still ongoing, then we would expect that:

- Prediction 1: *B*. *chinensis* will occur at high‐human visitation sites because these are locations where human‐mediated transport is most likely to overcome dispersal barriers. However, additional disturbance not related to human visitation rates (e.g., fire) should not make *B*. *chinensis* occurrence more likely than high‐visitation alone.
- Prediction 2: There will be differences in colony size based on time of colony establishment. In particular, supercolonies will be smaller at sites where *B*. *chinensis* has established more recently due to insufficient time to reach an equilibrium size.
- Prediction 3: Many regions in GSMNP and the southern Appalachians more broadly will be predicted suitable for *B*. *chinensis* , even though *B*. *chinensis* has not been found at these locations. These sites represent locations where *B*. *chinensis* has not yet dispersed but that could be occupied in the future.

Using our field and modeling results, we discuss the potential of *B*. *chinensis* to impact southern Appalachian ecosystems in the future, including impacts on the native ant species and on several ant‐dispersed plants. Ultimately, our goal is to lay the groundwork for future efforts to mitigate the effects of *B*. *chinensis* on the biodiversity harbored in GSMNP and in the southern Appalachians more broadly.

## 2 Methods

### 2.1 Data Collection

#### 2.1.1 Site Occupancy (Prediction 1)

**FIGURE 1:**
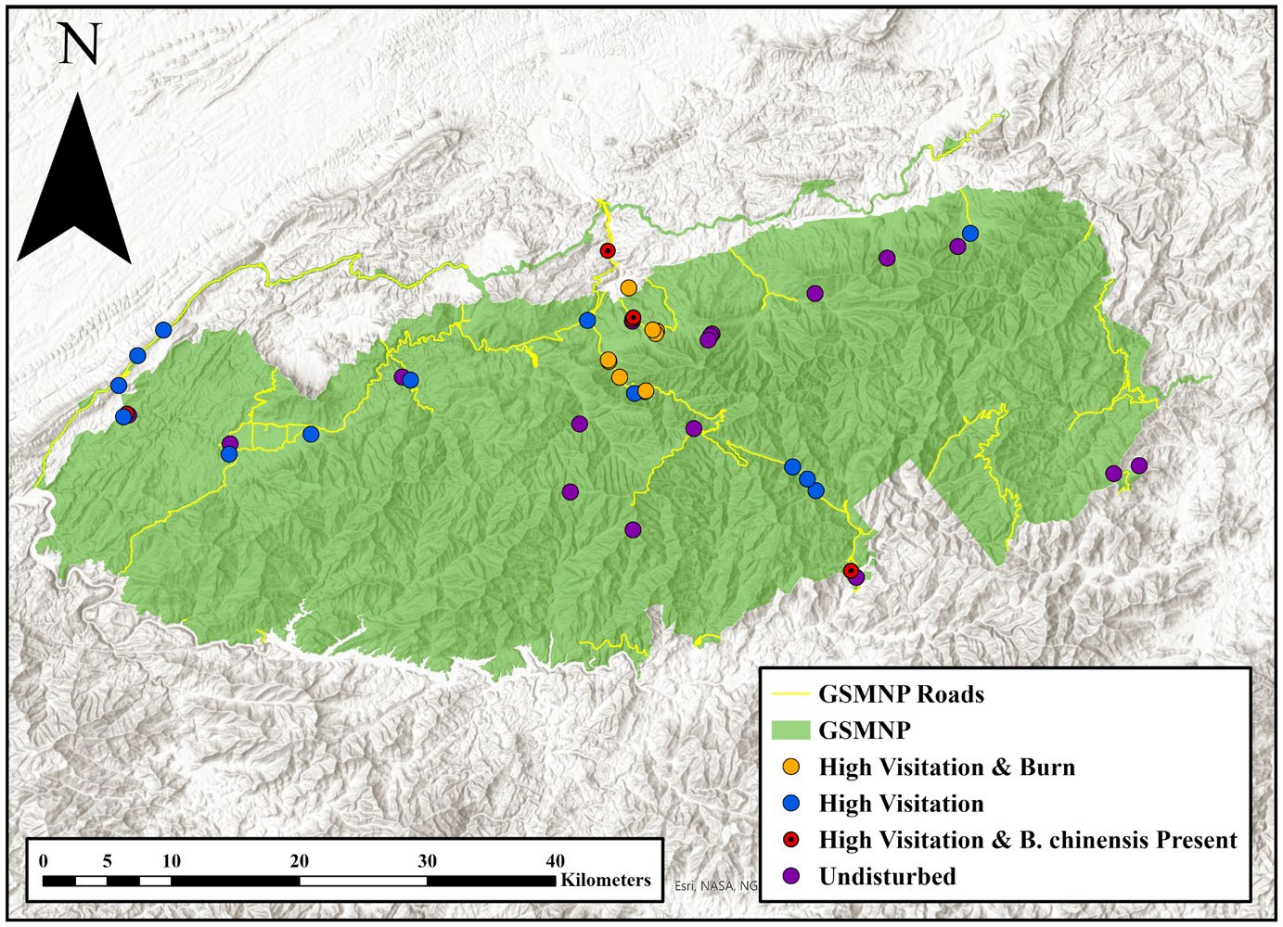
Map showing sampling locations classified by disturbance level and sites found to host *Brachyponera chinensis* across Great Smoky Mountains National Park.

We performed targeted surveillance for *B*. *chinensis* at *n* = 28 high visitation and/or high disturbance sites across GSMNP (Figure 1). Nineteen (*n* = 19) sites were chosen because they experience heavy human traffic (high visitation) and *n* = 9 were chosen because they were burned during the 2016 GSMNP fires (high “natural” disturbance, see Table 2). We selected high‐visitation sites based on their inclusion on the park website (https://www.nps.gov/grsm/planyourvisit/maps.htm) as places to see in GSMNP. We selected burn sites using the Avenza GSMNP Chimney Tops 2 Fire Burn Severity map (https://www.avenzamaps.com/maps/557998/great‐smoky‐mountains‐national‐park‐chimney‐tops‐2‐fire‐burn‐severity). Due to the challenges associated with accessing more remote burn sites on steep mountainsides with limited trails, most of our burn sites (*n* = 9 out of *n* = 10) were in locations that also experience heavy human traffic. Thus, almost all of our burn sites involved both high human visitation and high natural disturbance. For this reason, we focus on the contrast between high human visitation with and without “natural” disturbance due to fire for most of our analyses. Eight (*n* = 8) high visitation and *n* = 9 burn sites were sampled in both the spring (March 2021, *n* = 2–5 quadrats per site) and the summer (July 2021, *n* = 2–5 quadrats per site). Based on our findings from March an additional high visitation and burn site were added in the summer (July 2021, *n* = 2–5 quadrats per site). Finally, based on our findings from March and July, we returned to sample an additional *n* = 6 high disturbance sites in the Fall (October 2021, *n* = 2 quadrats per site) (Table 1; *n* = 130 samples total).

**TABLE 1:** Disturbed sampling locations with number of times visited and number of leaf litter samples.

Site	Site type	Times visited	Leaf litter samples
Abrams Creek	Undisturbed	8	39
Albright Grove	Undisturbed	8	40
Brushy Mtn	Undisturbed	8	39
Brushy Mtn Myrtle	Undisturbed	8	48
Cades Cove	Undisturbed	9	50
Cataloochee	Undisturbed	7	35
Double Springs Gap	Undisturbed	6	31
Elkmont	Undisturbed	8	39
Forney Ridge	Undisturbed	6	28
Indian Gap	Undisturbed	6	29
Oconaluftee	Undisturbed	8	15
Purchase Knob	Undisturbed	8	39
Ramsey Cascades	Undisturbed	7	43
Snakeden Ridge	Undisturbed	8	62
Treemont	Undisturbed	8	49
Trillium Gap	Undisturbed	8	39
Twin Creeks	Undisturbed	9	69

To compare *B*. *chinensis* occurrence at high visitation sites to *B*. *chinensis* occurrence at low visitation sites within GSMNP, we capitalized on our ongoing efforts to study ant communities across *N* = 16 sites that are in or adjacent to well‐characterized All Taxa Biodiversity Index (ATBI) plots (Nichols and Langdon 2007) as well as an additional site at one of the lowest elevations in the park (*n* = 17 total undisturbed; Table 1). These sites vary greatly in elevation (522–1975 m) and forest‐type and were selected to represent the dominant ecosystems present in the park. These sites receive far fewer visitors and are also significantly further away from GSMNP roads than high visitation sites (Figure S2). From August 2019 to April 2021, we collected between *n* = 4 and *n* = 15 quadrats of litter at 1 to 9 unique seasonal time‐points, depending on site accessibility (Table 2; *n* = 694 samples total).

**TABLE 2:** Undisturbed sampling locations with number of times visited and number of leaf litter samples.

Site	Site type	Times visited	Leaf litter samples
Abrams Creek Campground	High visitation	2	4
Abrams Creek Picnic Area	High visitation	2	4
Cades Cove Campground	High visitation	2	4
Cades Cove Visitor Center	High visitation	1	2
Collins Creek Picnic Area	High visitation	1	2
Cosby Campground	High visitation	2	4
Gatlinburg Welcome Center	High visitation	2	4
Kephart Prong Trailhead	High visitation	1	2
Look Rock A	High visitation	1	2
Mile Post 24.6 Parking Lot	High visitation	1	2
Oconaluftee Visitor Center	High visitation	2	4
Sugarlands Visitor Center	High visitation	2	4
Top of The World	High visitation	1	2
Treemont Disturbed	High visitation	2	4
Wolfpen Gap	High visitation	1	2
Carlos Campbell Overlook Unburnt	High visitation	2	7
Chimney Tops Picnic Unburnt	High visitation	2	7
Cove Hardwood Nature Trail	High visitation	1	5
Twin Creeks Unburnt	High visitation	2	7
Balsam Point Quiet Walkway	High visitaiton & burn	2	7
Baskin Creek Falls Trail	High visitaiton & burn	2	7
Baskin Creek Falls Trailhead	High visitaiton & burn	2	7
Carlos Campbell Overlook Low Burn	High visitaiton & burn	2	7
Carlos Campbell Overlook Med Burn	High visitaiton & burn	2	7
Chimney Tops Picnic Burnt	High visitaiton & burn	2	7
Gatlinburg	High visitaiton & burn	2	7
Twin Creeks Burn	High visitaiton & burn	2	7
Orchard Road near MM3	High visitaiton & burn	1	2

In addition to quadrat sampling, at most sites, we also spent an additional ~30 min searching for and aspirating ants during each visit. We primarily directed aspiration efforts toward surface collection (i.e., leaf surfaces, leaf litter, trees, bushes, and logs); however, we also overturned logs and rocks at each site to search for ants underneath. All sampling of both high visitation and low visitation sites took place during the daytime; however due to the volume of sites visited, we performed sampling at different times of day.

To collect ants from each 1 m × 1 m quadrat of litter we scraped the leaf litter and top ~1 cm of soil from each quadrat and sifted it to separate larger debris from fine litter and arthropods. We sifted until we saw no small debris left in the top aspect of the sifter and then placed all collected fine litter in a cotton bag along with a unique identifier. We tied bags at the top to prevent any arthropods from escaping and kept them moist until returning them to our lab for processing (typically *n* = 3–5 days). To extract litter arthropods, we hung samples in a temperature‐controlled laboratory using self‐assembled Winkler extractors (Ferro 2018) for *n* = 12 days and shook them *n* = 2 days after initial hanging to dislodge invertebrates trapped within the litter. Following storage in 100% ethanol, DAM, BTC, or AK identified ants, and *B*. *chinensis* identifications were later confirmed by Michael Ferro, director of the Clemson University Arthropod Collection. We conducted sampling under GSMNP permit number GRSM‐2020‐SCI‐2463.

#### 2.1.2 Supercolony Size (Prediction 2)

To assess the extent of the invasion at sites where *B*. *chinensis* was detected, either in litter sifts or via aspiration, we returned to the target locations in 2021 and 2022 and performed an exhaustive search for colonies and workers in the surrounding area. Specifically, searchers began at the original aspiration or leaf litter GPS coordinate and walked along the eight cardinal and intermediate directions from the GPS coordinate, marking coordinates wherever colonies or workers were found and continuing to move further until the invasion front was reached. Once we found no *B*. *chinensis* individuals after approximately 3 min of searching, we repeated the process in a different direction.

### 2.2 Data Analysis

#### 2.2.1 Site Occupancy (Prediction 1)

To gauge the degree to which human visitation alone versus human visitation in combination with natural disturbance (fire) facilitates the invasion of *B*. *chinensis* into GSMNP (i.e., the extent to which human‐aided dispersal versus habitat degradation is aiding *B*. *chinensis* establishment), we used one‐tailed Fisher's exact tests to determine whether the probability of occupancy of *B*. *chinensis* was higher in (1) burned high visitation versus unburned high visitation sites and (2) high visitation (burned and unburned) versus low visitation sites. (Because we did not find any difference based on burn status, we pooled burned and unburned human‐disturbed sites for the second analysis). We did not include the sole burn site that does not experience heavy human traffic for either of our comparisons. To assess the impact of access, rather than disturbance per se, we also conducted a “Near Analysis” relating *B*. *chinensis* occurrence to roadways using ArcGIS Pro's “Near” geoprocessing tool. Specifically, we estimated the mean distance of our sampling sites to major roads, for each of the following site types: (1) *B*. *chinensis* occurrence points, (2) uninvaded sites that experience heavy human traffic, (3) burn sites that experience heavy human traffic, and (4) sites that do not experience heavy human traffic. Again, we did not include the sole burn site that does not experience heavy human traffic for this analysis. We then performed a Kruskal–Wallis test and subsequent post hoc Dunn pairwise comparisons with Benjamini–Hochberg corrections in R 4.2.1 (R Core Team 2014) to compare means of all groups. We downloaded GSMNP road layer data from GSMNP's GIS page (https://grsm‐nps.opendata.arcgis.com/search?collection=Dataset&q=Transportation), and our R code and data are included in the Supporting Information.

#### 2.2.2 Supercolony Size (Prediction 2)

To quantify estimates of supercolony size we uploaded our within‐colony presence coordinates into ArcGIS Pro 2.8.7 for each site and used the “Aggregate Points” geoprocessing tool to create a buffered minimum convex polygon (MCP) for each invasion site. “Aggregate Points” produces an MCP by creating a polygon of the smallest possible area that contains all the coordinates in the dataset. MCP's are often used to estimate home range size of populations (Silvy 1960). Creating an MCP at each of our invaded sites allowed us to estimate the area that each population has spread and determine an average area of invasion for each supercolony.

#### 2.2.3 Habitat Suitability (Prediction 3)

We created two maximum entropy (Maxent) SDMs using our own observations and existing records. We created one *B*. *chinensis* SDM using only North American invasion presences (*n* = 67) and a second SDM using presence points from the *B*. *chinensis* native and invasive ranges (*n* = 254). *Brachyponera chinensis* presence data were generously provided from previous *B*. *chinensis* SDM efforts (Bertelsmeier, Guénard, and Courchamp 2013), manually extracted from the AntWeb, Global Ant Biodiversity Informatics (Guénard et al. 2016), iNaturalist databases or first collected here. iNaturalist presences were filtered to include only “Research Grade” observations with greater than 2 agreements and 0 disagreements and were further verified by DAM based on visual inspection. We filtered downloaded data based on a variety of criteria meant to increase the probability of accurate identifications. Specifically, we included presence data in our analyses only if accompanied by GPS coordinates, collection dates, collector ID, and associated publication citation or museum catalog number. Presence data and associated metadata can be found in the Supporting Information.

SDMs were built using SDMtoolbox (Brown 2014) in ArcGIS Pro and Maxent (Phillips et al. 2017). SDMtoolbox is a python‐based GIS toolbox which preprocesses bioclimatic data and creates executable batch scripts and input files for Maxent. We downloaded bioclimatic data and elevation data from the WorldClim (Fick and Hijmans 2017) database at a resolution of 30 Arc‐seconds (~1 km^2^). For bioclimatic data, we used six variables as follows: BIO1 (annual mean temperature), BIO2 (mean diurnal range), BIO6 (minimum temperature of coldest month), BIO8 (mean temperature of wettest quarter), BIO11 (mean temperature of coldest quarter), and BIO19 (precipitation of coldest quarter) (Fick and Hijmans 2017). We selected these bioclimatic variables as they are the most relevant to *B*. *chinensis* based off previous literature on species natural history (Bertelsmeier, Guénard, and Courchamp 2013). We did not attempt to pick independent predictor variables, because collinearity does not significantly influence Maxent model predictions (Alsamadisi, Tran, and Papeş 2020). We spatially rarified presence data at a resolution of 1 km^2^ to reduce spatial autocorrelation. We created a bias file for Maxent projection using background selection of points limited to an area encompassed by a buffered minimum‐convex polygon based on observed localities with a buffer distance of 200 km. We tested Maxent models via 25% random test percentage subsampling. We used 10,000 background points, a regularization multiplier of 3, and a maximum convergence of 5000 iterations with ten percentile training presence for model building. We ran 25 replicates of the above model and averaged test data omission rates, test data average area under the cover (AUC), and overall pixel suitability for our SDMs. We evaluated conditional statistics (e.g., maximum elevation where *B*. *chinensis* is at least 50% likely to be found) using the “con” function in ArcGIS Pro geoprocessing for our GSMNP and southeastern spatial scales.

To investigate differences in precipitation at high‐elevation occurrences of *B*. *chinensis* between their native range and GSMNP, we imported rasters for elevation (m), annual precipitation (mm; BIO12), and annual precipitation of the coldest quarter (mm; BIO19) from the WorldClim database into ArcGIS Pro at a 30 Arc‐second (~1 km^2^) resolution. We then imported occurrence records of *B*. *chinensis* from their native range and GSMNP as xy‐point data and used the “Extract Values to Points” Geoprocessing tool to assign elevation and precipitation values to each occurrence point. We then assembled these data into a single .csv file after extracting them from ArcGIS Pro using the “Table To Excel” tool and calculated average annual precipitation and average annual precipitation of coldest quarter across high‐elevation *B*. *chinensis* occurrences within their native range and GSMNP. We additionally compared annual precipitation of the coldest quarter between native and GSMNP‐invasive *B*. *chinensis* along a high‐elevation gradient using a generalized linear model (GLM) following a Poisson distribution.

#### 2.2.4 Impact on Native Species

To determine the habitat suitability of *A*. *rudis* *s*.*l*. in both GSMNP and the southeastern U.S. more broadly, we downloaded presences across North America from the AntWeb database or first published here to create a separate *A*. *rudis* *s*.*l*. SDM (*n* = 414). We filtered downloaded data based on a variety of criteria meant to increase the probability of accurate identifications (see previous section for details). We did not include records from iNaturalist because of the difficulty of identifying *Aphaenogaster* spp. and inconsistent location accuracies. Presence data included in this manuscript and associated metadata can be found in the Supporting Information.

To build *A*. *rudis* refugia models, we clipped *B*. *chinensis* and *A*. *rudis* *s*.*l*. SDMs to the extent of GSMNP using the “Clip Raster” function in ArcGIS Pro's Geoprocessing tool, allowing us to calculate SDM spatial statistics for GSMNP and the southeastern U.S. separately. After clipping, we overlaid the SDMs for *A*. *rudis* and *B*. *chinensis* with the “Raster Calculator” function in ArcGIS Pro's Geoprocessing tool; this allowed us to combine the two SDM outputs for every 1km^2^ grid of GSMNP. From this, we developed a refugia model as follows:

$$P\left(A.\;\text{rudis}\;s.l.\;\text{refugia}\right)=\left(1-P\left(B.\;\text{chinensis}\;\right)\right)\times P\left(A.\;\text{rudis}\;s.l.\;\right)$$

This model calculated the joint probability of any grid square simultaneously being unsuitable for *B*. *chinensis* but suitable for *A*. *rudis* *s*.*l*. Since *B*. *chinensis* SDMs constructed from invasive versus invasive and native points predicted similar suitabilities (see results below), for modelin*g Aphaenogaster* refugia we used the slightly more conservative *B*. *chinensis* SDM constructed from both invasive and native presences.

Because *B*. *chinensis* can disrupt ant‐plant mutualisms (Rodriguez‐Cabal et al. 2012) vital for myrmecochorous plant fecundity and dispersal, we created joint myrmecochorous plant and *A*. *rudis* *s*.*l*. refugia models for five myrmecochorous species found within GSMNP. For this, we used previous GSMNP myrmecochorous plant SDMs (Stark and Fridley 2022) for *Viola* and *Sanguinaria*. We created the *Trillium* spp. SDMs using iNaturalist observations. iNaturalist presences were filtered to include only “Research Grade” observations with greater than 2 agreements and 0 disagreements and were further verified by DAM. We overlaid SDMs with the “Raster Calculator” Geoprocessing tool in ArcGIS Pro; this allowed us to combine both *A*. *rudis* *s*.*l*. refugia outputs and five myrmecochorous plant SDMs— *Sanguinaria canadensis* , *Viola rotundifolia* , *Viola sororia* , *Trillium simile* , and *Trillium vaseyi—*for every 1 km^2^ grid of GSMNP. From this, we developed five refugia models as follows:

$$P\left(S.\;\text{canadensis refugia}\right)=P\left(S.\;\text{canadensis}\right)\times P\left(A.\;\text{rudis}\;s.l.\;\text{refugia}\right)$$

$$P\left(V.\;\text{rotundifolia refugia}\right)=P\left(V.\;\text{rotundiflora}\right)\times P\left(A.\;\text{rudis}\;s.l.\;\text{refugia}\right)$$

$$P\left(V.\;\text{sororia refugia}\right)=P\left(V.\;\text{sororia}\right)\times P\left(A.\;\text{rudis}\;s.l.\;\text{refugia}\right)$$

$$P\left(T.\;\text{simile refugia}\right)=P\left(T.\;\text{simile}\right)\times P\left(A.\;\text{rudis}\;s.l.\;\text{refugia}\right)$$

$$P\left(T.\;\text{vaseyi refugia}\right)=P\left(T.\;\text{vaseyi}\right)\times P\left(A.\;\text{rudis}\;s.l.\;\text{refugia}\right)$$

These models calculated the joint probability of any grid square simultaneously being a refuge for *A*. *rudis* *s*.*l*. and being suitable for each myrmecochorous species.

For the *B*. *chinensis* and *A*. *rudis* *s*.*l*. SDMs and for the *A*. *rudis* *s*.*l*. refugia model, we calculated the following statistics at both the GSMNP and the southeastern U.S. spatial scales (southeastern states = TN, SC, NC, AL, GA, FL, KY, VA): mean percent suitability and percent of area with > 50% probability.

For the GSMNP myrmecochorous refugia models we calculated the following statistics at both the GSMNP and the southeastern U.S. spatial scales: mean and median percent refugia and percent of area with > 50% probability of being a refugium.

## 3 Results

### 3.1 Site Occupancy (Prediction 1)

We found *B*. *chinensis* at *n* = 4 of *n* = 15 (unburned) high‐visitation sites (the Abrams Creek Campground, the Oconaluftee Visitor Center, the unburned area around Twin Creeks Science and Education Center, and the Gatlinburg Welcome Center), and *n* = 1 of *n* = 9 high visitation burn sites (the burn area around Twin Creeks Science and Education Center). We did not find *B*. *chinensis* at any of the *n* = 17 low visitation unburned sites nor did we find *B*. *chinensis* at the *n* = 1 low visitation burn site (Figure 1). Thus, all *n* = 5 sites where we found *B*. *chinensis* , including the single burn site where the species is established, are high‐visitation areas. However, we did not find a significant difference in *B*. *chinensis* occupancy in burned versus unburned high visitation sites (Fisher's exact test *p*‐value: 0.3134) and the difference in *B*. *chinensis* occupancy in high visitation versus low visitation sites was only borderline significant (Fisher's exact test *p*‐value: 0.0866; for Near Analysis, see Figure S2).

### 3.2 Supercolony Size (Prediction 2)

**FIGURE 2:**
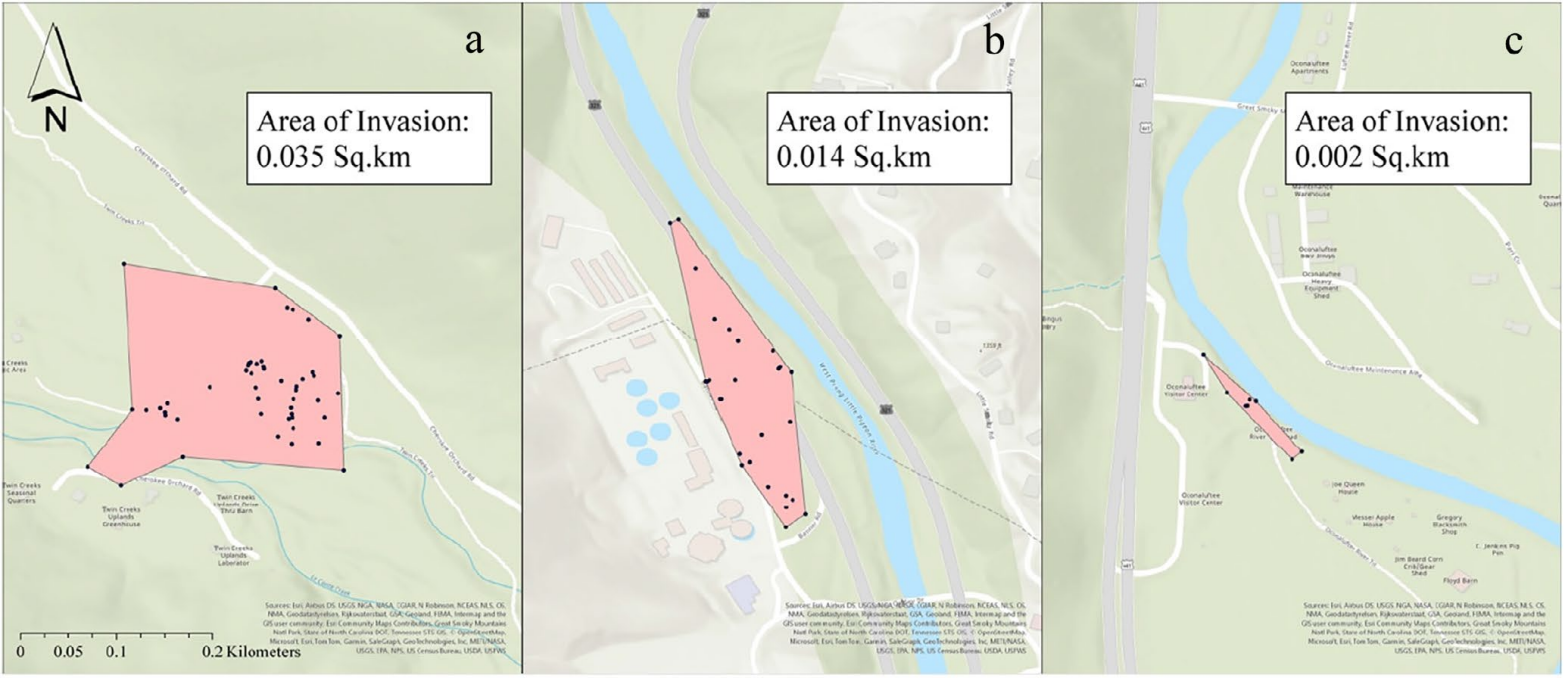
Three multiyear supercolonies found within GSMNP at (a) Twin Creeks Science Center, (b) Gatlinburg Welcome Center, and (c) Oconaluftee Visitor Center. Mean size of supercolonies: 0.017 km^2^. Blue dots represent individual presence points of *Brachyponera chinensis* and red polygons represent estimated extent of supercolonies.

We were only able to relocate workers at *n* = 4 of the *n* = 5 sites where we initially detected *B*. *chinensis* . At the Abrams Creek Campground, continued search efforts failed to detect additional colonies or foraging workers. Given that our only record at this location was a few ants found in a single litter extraction, we assume that *B*. *chinensis* at the Abrams Creek Campground either failed to establish or exists as a new and relatively small incipient colony. Additional surveillance in this area is highly recommended. At the remaining four sites where we found *B*. *chinensis* , estimated supercolony sizes ranged from 1783 to 34,514 m^2^ (Figure 2). Interestingly, the smallest supercolony was at the Oconaluftee Visitor Center. To our knowledge, this is the only site where *B*. *chinensis* has been previously recorded within park boundaries (all other foothills locations are outside the park), and thus likely represents one of the oldest invasions (> 15 years) (Paysen 2007). We found the largest supercolony at the Twin Creeks Science Center. Here, the supercolony occurred over a contiguous region encompassing both one of our burned high‐visitation sites and one of our unburned high‐visitation sites. Because the invasion at this location spanned two of our sites, we treated it as a single invasion location for all subsequent analysis. Mean supercolony size across all three invasion locations was 16,913 m^2^.

### 3.3 Habitat Suitability (Prediction 3)

**FIGURE 3:**
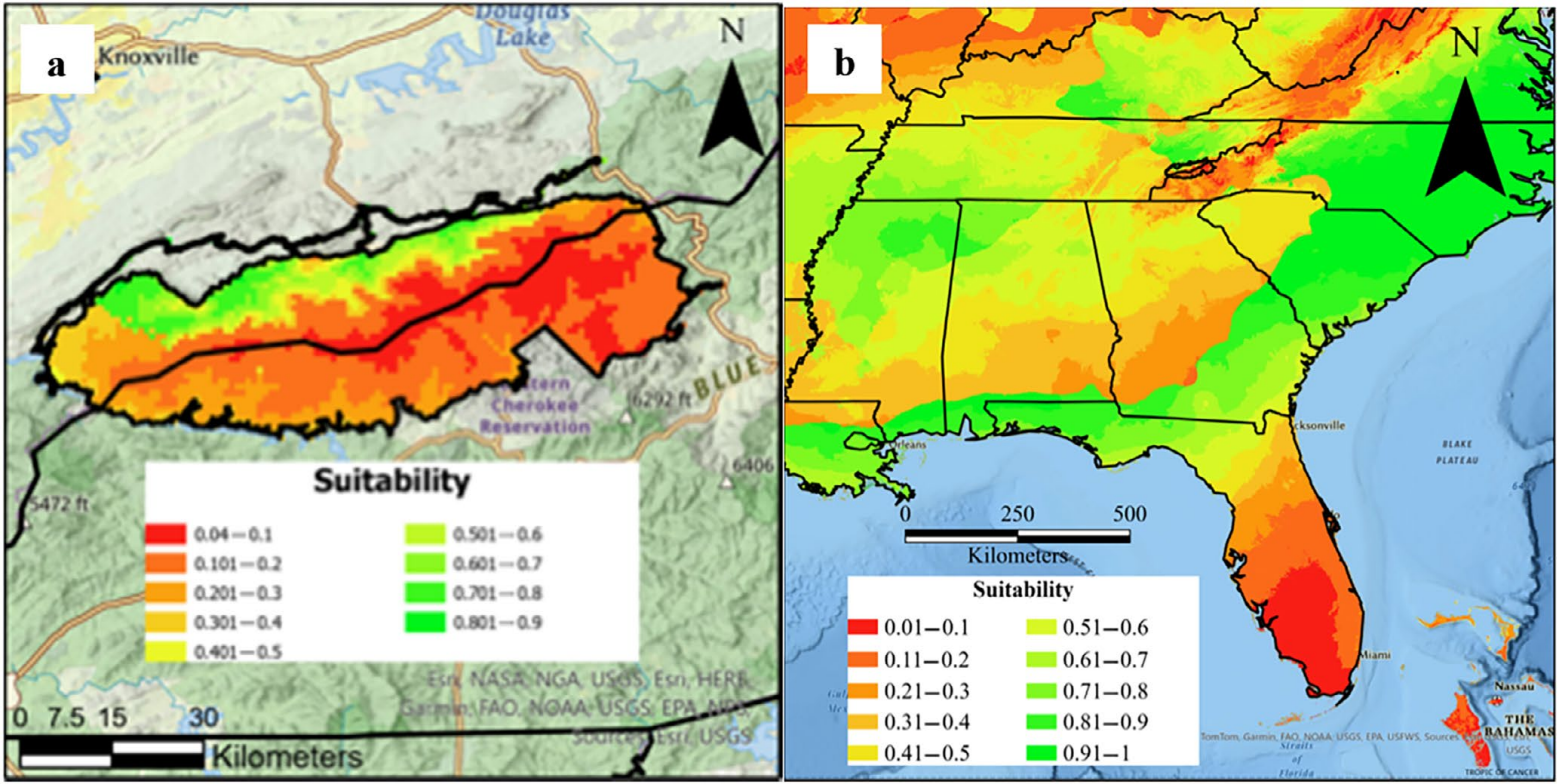
Estimated probability of *Brachyponera chinensis* suitability (using Global SDM) at a 1 km^2^ resolution in (a) GSMNP and (b) the southeastern U.S.

*Brachyponera chinensis* SDMs constructed from solely invasive versus invasive and native records predicted similar suitabilities across both GSMNP and the southeastern U.S. (Figure 3). Models using only invasive presences (*n* = 67) predicted moderate suitability across the southeastern U.S. (mean suitability is 0.55; Supporting Information), as well as moderate suitability across GSMNP (mean suitability is 0.31; Supporting Information), with suitability across GSMNP lower than suitability across the southeastern U.S. more broadly. Overall, these models predicted that 19% of GSMNP and 54% of the southeastern U.S. had a greater than 50% chance of being suitable for *B*. *chinensis* . Models using both native and invasive presences (*n* = 254) similarly predicted moderate suitability across the southeastern U.S. (mean suitability is 0.57), as well as moderate suitability across GSMNP (mean suitability is 0.27). Again, suitability was lower in GSMNP as compared to the southeastern U.S. Overall, 19% of GSMNP and 53% of the southeastern U.S. had a greater than 50% chance of being suitable for *B*. *chinensis* . In all models, the low elevations along the northern border of the park were more suitable than the rest of GSMNP. Likewise, the low‐elevation western portion of the park was more suitable than the high‐elevation eastern portion. Interestingly, however, neither temperature nor elevation were strong predictors in any of the *B*. *chinensis* models that we tested, with percent contributions ranging from 0.5% to 19.2%, depending on the model and the temperature/elevation variable considered. By contrast, the greatest percent contribution (69.1%–70.4%) was from the BIO19, which reflects precipitation during the coldest quarter. This suggests that precipitation at times of low temperature, rather than temperature itself, underlies the resilience of high‐elevation southern Appalachian communities to *B*. *chinensis* invasion (for results comparing high‐elevation precipitation regimes in native and invasive ranges see Figure S1).

### 3.4 Impact on Native Species

**FIGURE 4:**
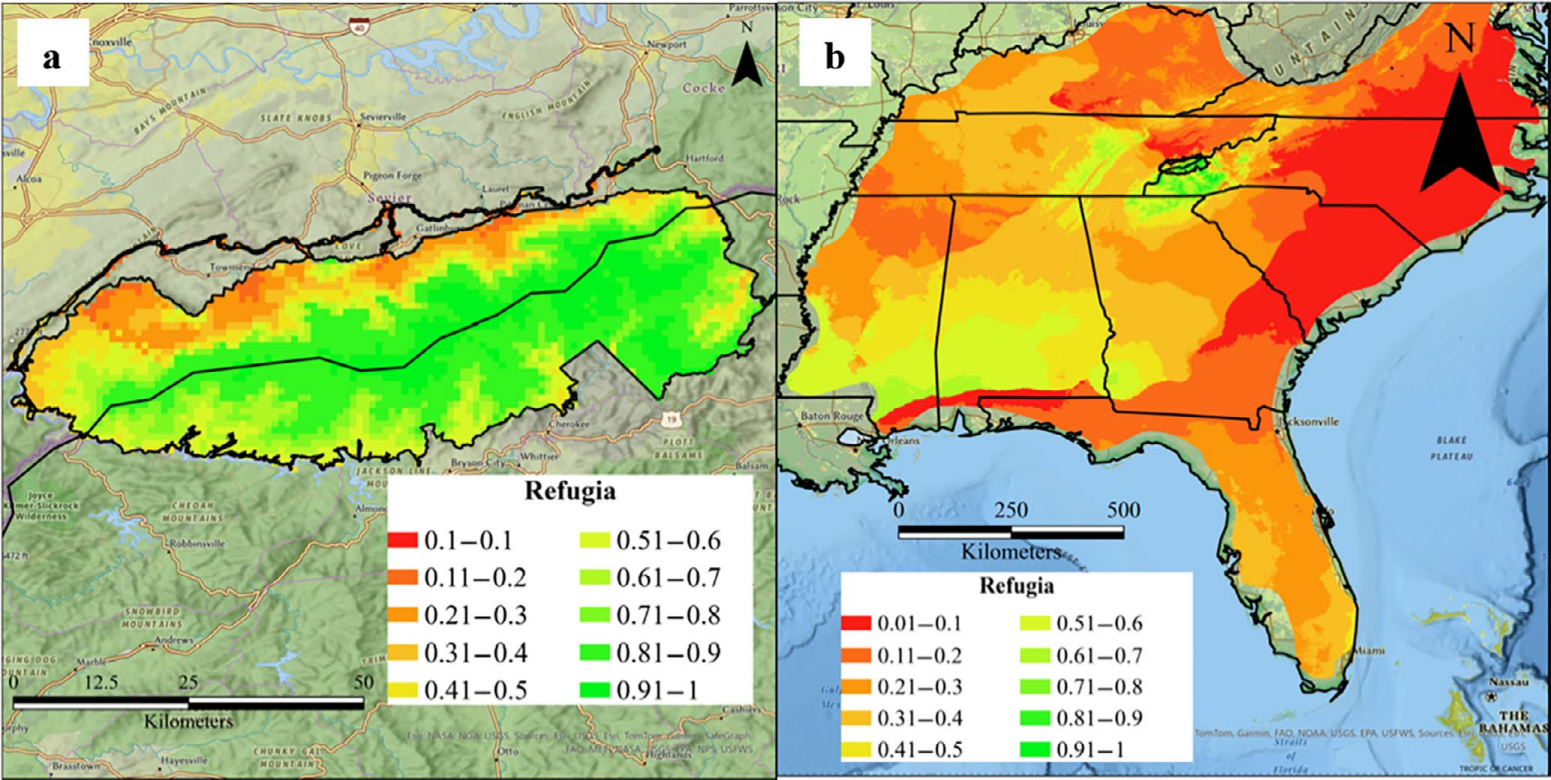
Refugia model of *Aphaenogaster rudis sensu lato* in (a) GSMNP and (b) southeastern U.S. comprised of two overlaid SDMs from *Brachyponera chinensis* and *A*. *rudis* *s*.*l*.

*Aphaenogaster rudis s*.*l*. Maxent models predicted high suitability across GSMNP (mean = 0.89) and moderate suitability across the southeastern U.S. (mean = 0.59). Ninety‐nine percent of GSMNP and 50% of the southeastern U.S. have a greater than 50% chance of being suitable for *A*. *rudis* *s*.*l*. Notably, refugia probabilities for *A*. *rudis* *s*.*l*. in GSMNP are dramatically higher than they are across the southeastern U.S. overall. Mean and median probabilities of *A*. *rudis* *s*.*l*. refugia in GSMNP are 0.67 and 0.75 (Figure 4A). Approximately 75% of the park has a greater than 50% chance of classifying as refugia for *A*. *rudis* *s*.*l*. By contrast, mean and median probabilities of *A*. *rudis* *s*.*l*. refugia in the southeastern U.S. are 0.31 and 0.22 (Figure 4B). Approximately 8% of the southeastern U.S. has a greater than 50% chance of classifying as *A*. *rudis* *s*.*l*. refugia.

**FIGURE 5:**
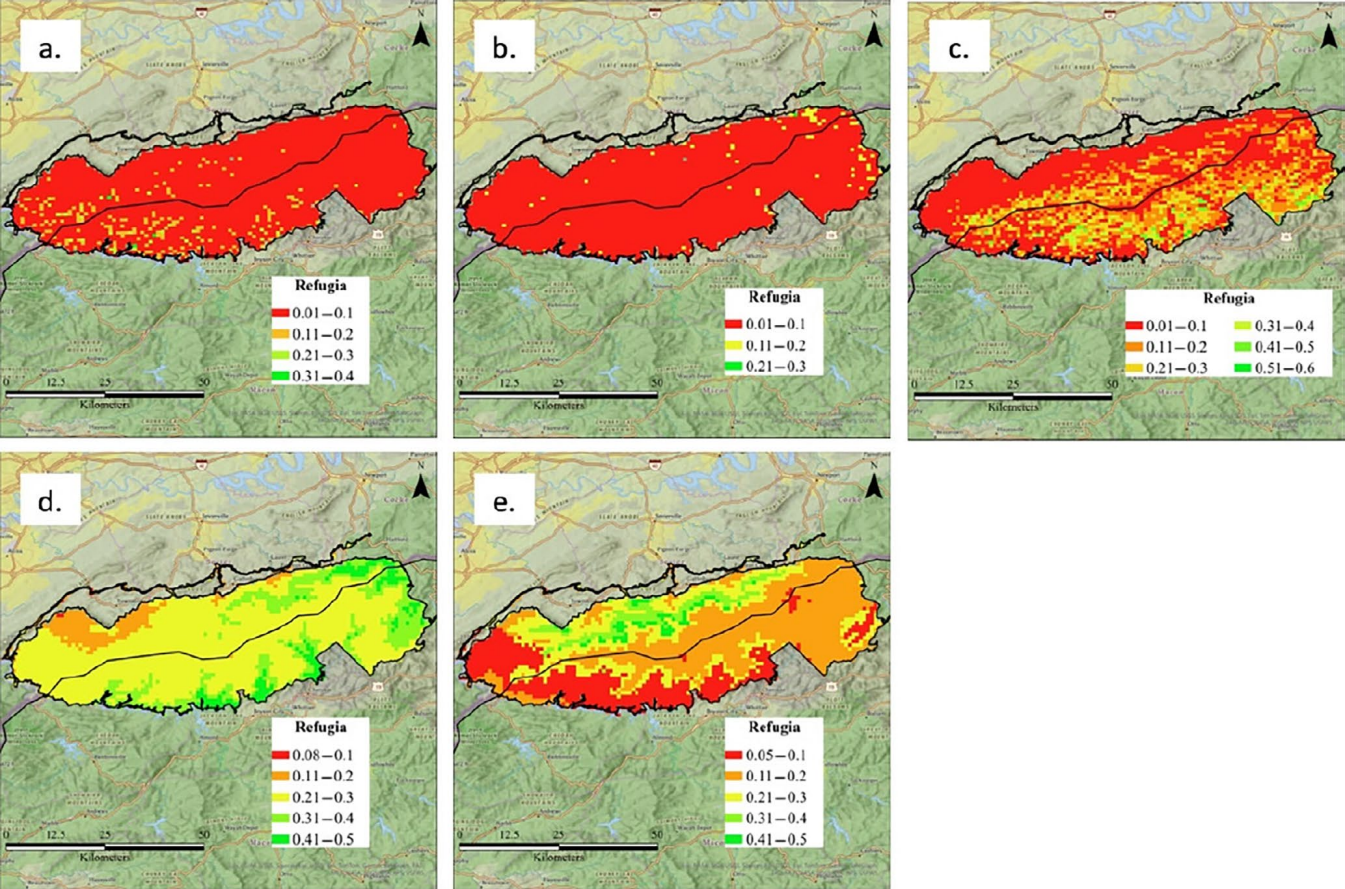
GSMNP Refugia models of (a) *Sanguinaria canadensis* , (b) *Viola sororia* , (c) *Viola rotundifolia* , (d) *Trillium vaseyi* , and (e) *Trillium simile* . All refugia models are comprised of two stacked SDMs, with the first being an SDM of plant suitability and the second being an SDM of *Aphaenogaster rudis sensu lato* refugia probability.

Mean and median probabilities of *S*. *canadensis* refugia in GSMNP are 0.03 and 0.02 (Figure 5A). None of the park has a greater than 50% chance of classifying as suitable refugia for *S*. *canadensis* . Mean and median probabilities of *V*. *sororia* refugia in GSMNP are 0.02 and 0.01 (Figure 5B). None of the park has a greater than 50% chance of classifying as suitable refugia for *V*. *sororia* . Mean and median probabilities of *V*. *rotundifolia* refugia are 0.11 and 0.08 (Figure 5C). Less than 1% of the park (area = 21.14 km^2^) has a greater than 50% chance of classifying as suitable refugia for *V*. *rotundifolia* . Mean and median probabilities of *T*. *simile* refugia in GSMNP are 0.175 and 0.17 (Figure 5D). None of the park has a greater than 50% chance of classifying as suitable refugia for *T*. *simile* . Mean and median probabilities of *T*. *vaseyi* refugia in GSMNP are 0.26 and 0.24 (Figure 5E). None of the park has a greater than 50% chance of classifying as suitable refugia for *T*. *vaseyi* .

## 4 Discussion

The goal of this study was to assess the current and future potential of *B*. *chinensis* to invade GSMNP and the southern Appalachians more broadly. In particular, we sought to determine whether the limited spread of *B*. *chinensis* into high‐elevation GSMNP and southern Appalachian ecosystems is a result of unsuitable habitat or inadequate time to reach these less accessible locations. To address these questions, we characterized the role of human visitation with and without additional “natural” disturbance (fire) on *B*. *chinensis* occurrence. We also estimated supercolony sizes at long‐standing and likely newer sites of invasion. Finally, we used our own and existing *B*. *chinensis* occurrence records to predict suitable habitats of *B*. *chinensis* across GSMNP and the southeastern U.S. Previously, *B*. *chinensis* had only been found at a single location within the park (Paysen 2007). Our efforts uncovered three additional GSMNP sites (four if both locations at Twin Creeks Science Center are counted separately, see above). Fortunately, none of these sites were in intact forest, suggesting that pristine habitats within the park, even at lower elevations, may not have yet been colonized.

### 4.1 Evidence for Ongoing Invasion

Our finding that *B*. *chinensis* is preferentially associated with high visitation sites and not currently found in intact forest runs counter to existing literature on the broader invaded range of *B*. *chinensis* across the southeastern U.S., where supercolonies have widely been found to establish successfully in disturbed areas and intact forests alike (Guénard, Wetterer, and Macgown 2018; Guénard and Dunn 2010). This difference can, however, be explained if occurrences in GSMNP reflect dispersal barriers rather than habitat suitability. In this case, *B*. *chinensis* is less likely to penetrate intact forests, not because it cannot successfully establish at forested sites, but because it is less likely to reach these sites. Our finding that high human visitation combined with natural (fire) disturbance does not greatly increase occurrence probability is in line with such a scenario. If disturbance and/or a degraded canopy strongly aided in *B*. *chinensis* establishment, then sites with high visitation *and* burn should be significantly more invaded, which that is not what we observe. Thus, we interpret our findings as evidence that human dispersal is likely facilitating early invasion into the park (Campbell et al. 2019; Warren et al. 2020). Unfortunately, this suggests that the invasion may not yet have reached equilibrium and that *B*. *chinensis* may eventually penetrate the intact forests of GSMNP. Consistent with the suggestion that *B*. *chinensis* may eventually penetrate intact forests in GSMNP, SDMs predicted a broad region of suitable habitat along the northern border of the park (Figure 4), including a number of sites where we searched for but did not find *B*. *chinensis* colonies.

### 4.2 Evidence for Abiotic and/or Biotic Resistance to Invasion

Interestingly, the regions where SDMs predicted high suitability are also the regions where we found our two largest *B*. *chinensis* supercolonies. By contrast, SDMs predicted low suitability at Abrams Creek Campground and Oconaluftee Visitor Center, where we found small and/or incipient *B*. *chinensis* colonies. At the Abrams Creek Campground, the invasion seems to be halted or in an early stage, as it was never found more than once despite extensive sampling. At the Oconaluftee Visitor Center, which has been colonized for at least 15 years, the supercolony is still relatively small. To our knowledge, no studies have characterized the rate of colony expansion of *B*. *chinensis* , and few studies have characterized rates of colony expansion of invasive ants in general (Tartally 2006; Abbott 2006). Nevertheless, the small size of the *B*. *chinensis* colony at the Oconaluftee Visitor Center relative to the sizes of the supercolonies at sites along the northern border suggests that the supercolony at the Oconaluftee Visitor Center is spreading slowly. The introduction at this site is likely older than the introductions at the Twin Creeks Science Center and the Gatlinburg Visitor Center. Thus, if the invasion was strictly limited by dispersal, we would expect the colony at Oconaluftee Visitor Center to be the largest. The fact that it is actually the smallest suggests that biotic and abiotic barriers not present along the northern border may be limiting invasion (Menke and Holway 2006; Levine, Adler, and Yelenik 2004; Warren et al. 2020) along the southern border. This is consistent with our SDMs that predict much lower habitat suitability for *B*. *chinensis* at both the Abrams Creek Campground and the Oconaluftee Visitor Center. While dispersal may be limiting *B*. *chinensis* in some regions of the park, there appear to be environmental barriers to spread throughout other regions of the park. Further supporting the claim that dispersal limitation cannot fully explain the absence of *B*. *chinensis* in GSMNP, our SDMs suggest that a significant portion of the park is minimally suitable for *B*. *chinensis* . Notably, even in the two areas of lower suitability where we found incipient and slowly spreading *B*. *chinensis* colonies, the probability of colonization was still > 20%. Much of the park has even lower probabilities of colonization, and we did not find any *B*. *chinensis* colonies in these regions. Fortunately, many of the park's regions least likely to be colonized by *B*. *chinensis* are the sensitive high‐elevation ecosystems that harbor exceptional endemism.

### 4.3 Abiotic Factors Preventing Invasion

Our finding that high elevations in GSMNP are largely unsuitable for *B*. *chinensis* is consistent with previous findings that *B*. *chinensis* is unlikely to colonize high elevations across its invasive range (Warren et al. 2020). However, whereas previous studies have suggested that temperature might render high‐elevation sites uninhabitable (Warren et al. 2020), our SDMs indicate that temperature variables alone, at least at the scale and resolution that we consider (i.e., across the southern Appalachians), have a relatively small impact on *B*. *chinensis* habitat suitability. Rather, *B*. *chinensis* habitat suitability appears to be determined by the combined effect of temperature and precipitation (see Supporting Information zipped file for analysis of variable contributions). This agrees with a previous study of *B*. *chinensis* that found large contributions to suitability from precipitation variables (Bertelsmeier, Guénard, and Courchamp 2013); however, these models were built at a much coarser resolution (18 km × 18.5 km). Thus, they addressed geographic extent, but not the penetrability of high‐elevation ecosystems in the southern Appalachians. By contrast, our highly resolved (1 km × 1 km) SDMs provide a more nuanced picture of the *B*. *chinensis* invasion in this region.

It is not surprising that previous studies have associated elevation and, by extension, temperature, with resistance to *B*. *chinensis* across its invasive range. Throughout the southern Appalachians, both precipitation and temperature co‐vary predictably along elevational gradients, at least at coarse spatial resolutions. More specifically, precipitation increases with elevation, while temperature decreases (Meiners et al. 1984; Bolstad, Vose, and McNulty 2001; Bolstad and Vose 2001; Smallshaw 1953). This collinearity can make it difficult to disentangle the effects of temperature versus precipitation. However, when using analyses at finer spatial resolutions, for example, at spatial resolutions that enable resolved slope‐to‐slope comparisons, the collinearity between temperature, precipitation, and elevation often breaks down (Körner 2007). For instance, north‐ versus south‐facing slopes (Reid 1973) and leeward versus windward slopes (Konrad 1994) typically receive different precipitation at the same elevation and temperature. Likewise, prevailing winds can cause regions of a mountain range to experience different precipitation regimes (Konrad 1994). This is the case for our study region. More specifically, given the complex topology across much of the southern Appalachians, there is a breakdown in collinearity between precipitation, temperature, and elevation at resolutions that make individual slopes distinguishable. There is, for example, a southwest‐to‐northeast gradient of decreasing precipitation (Wiser, Peet, and White 1996). This gradient emerges because most precipitation comes from southwesterly winds (Dickson 1959), which results in the southern side of GSMNP receiving more precipitation than the northern side, even at similar elevations. High precipitation along the southern border of GSMNP explains the lower *B*. *chinensis* suitability in this region of the park. It also explains the much higher *B*. *chinensis* suitability along the northern border of the park. We capitalized on these small‐scale features by creating highly resolved SDMs (1 km × 1 km). Granted our SDMs were at a spatial resolution that preserves natural, slope‐to‐slope disruptions to the collinearity between precipitation, temperature, and elevation, we were able to attribute *B*. *chinensis* habitat suitability to the combined effect of precipitation and temperature, rather than temperature alone.

Our conclusion that precipitation is a likely driver of *B*. *chinensis* habitat suitability in GSMNP manifests itself in the fact that our models predict low suitability at high elevations across the park, where precipitation is relatively high. These findings are noteworthy in comparison to occurrence records from the native range of *B*. *chinensis* , where colonies have been found inhabiting much higher elevations than our models predict suitably. This is likely due to the fact that most high‐elevation occurrences in the *B*. *chinensis* native range receive far less precipitation during the coldest quarter of the year than high‐elevation regions in the southern Appalachians (Figure S1). This is not surprising given some high‐elevation southern Appalachian ecosystems are temperate rainforests, receiving some of the highest rainfall in the eastern U.S (Henson, Kolawole, and Ayeni 2014). Such differences in bioclimatic conditions reinforce the risks of using presence data within a species' native range for the generation of SDMs for its invasive range, in line with the findings of other studies (Mainali et al. 2015; Barbet‐Massin et al. 2018).

### 4.4 Effects on Native Species

One of the main threats of *B*. *chinensis* is its ability to outcompete and extirpate native ant species such as *A*. *rudis* *s*.*l* (Warren et al. 2020). Unfortunately, our joint SDM models indicate that only a small percentage (9%) of the southeastern U.S. is likely to serve as *A*. *rudis* *s*.*l*. refugia. In this sense, GSMNP and the southern Appalachian regions directly south of GSMNP (e.g., Pisgah National Forest, Cherokee National Forest, and Nantahala National Forest) are important conservation areas for *A*. *rudis* *s*.*l*. (Figure 5) because these are the only areas across the entire southeastern U.S. where *A*. *rudis* *s*.*l*. is likely to avoid *B*. *chinensis* . Conservation of *A*. *rudis* *s*.*l*. is important because of its role as the dominant seed‐disperser of myrmecochores (Rodriguez‐Cabal et al. 2012; Buono et al. 2022; Ness, Morin, and Giladi 2009). This, however, will be of little benefit if *A*. *rudis* *s*.*l*. only persists in refugia outside myrmecochore ranges. Unfortunately, our preliminary analysis suggests that many of the areas likely to serve as *A*. *rudis* *s*.*l*. refugia are not habitable by at least five common myrmecochorous plants, including two endemic to the southern Appalachians (Figure 5). Rather, for the spring ephemeral species that we considered, suitable habitat tends to be in drier, low‐elevation regions where *B*. *chinensis* is most likely to invade. Whether habitat suitability is similar across the majority of other myrmecochorous plant species remains unknown.

### 4.5 Caveats and Future Directions

Our study presents preliminary findings on the current extent and future potential of *B*. *chinensis* to invade GSMNP and the southern Appalachians, yet there are nevertheless limitations to our results. These limitations should be considered when assessing our major findings and when developing future research and management extensions to the current study. First, our conclusions are heavily based on *in silico* results from MaxEnt models. This allows us to project areas at risk of *B*. *chinensis* invasion before they are invaded. However, like all models, our results are strongly dependent on model input and model assumptions. Model results could, for instance, change with additional occurrence records in either the native or invasive ranges. Model results could also change if different environmental variables are considered. In addition, model results depend on all of the standard assumptions of MaxEnt models, for example, that species distributions are largely driven by environmental conditions rather than species interactions. One way to deal with the uncertainty of our *in silico* models is to ground‐truth them using field data. Unfortunately, this is difficult for invasive species where a lack of occurrence at sites of predicted high suitability may reflect poor model fit, but could just as easily reflect our perspective that invasion is a dynamic process and that the invasion under investigation has not yet reached equilibrium. Despite this challenge, our conclusions would benefit from additional on‐the‐ground validation, both for *B*. *chinensis* and for the native species that we consider. Verifying our predictions through increased search efforts both inside and outside of predicted high suitability habitat, as well as through measurement of additional microclimate variables (Abbott 2006; Menke and Holway 2006) such as soil moisture, rates of evapotranspiration, and drainage at sites of infestation would help to both validate and refine our models. A second limitation of our study is that our models of future invasion potential assume current climate conditions. However, if invasion is relatively slow, then the *B*. *chinensis* invasion could be impacted by ongoing climate change. An interesting extension of our work would be to perform a similar analysis with projections of future climatic conditions at higher resolutions (previous SDMs did project into the future, but are limited in their applicability to GSMNP by their resolution) in our study area of interest. In particular, future work might determine whether climate change increases or decreases the invasion potential of *B*. *chinensis* in GSMNP and the ensuing refugia of *Aphaenogaster* spp. and their myrmecochorous plants.

Despite the limitations of our study, we present the most detailed current perspective on how *B*. *chinensis* may impact GSMNP. Our results highlight the differential risk of *B*. *chinensis* invasion in different regions of the park. Thus, similar to SDM models of other invasive species (Meriggi et al. 2022; Pěknicová and Berchová‐Bímová 2016; Bazzichetto et al. 2018), our results can be used as a tool for guiding early detection and monitoring of *B*. *chinensis* within the park. Even if these predictions are imperfect, they can be useful for improving *B*. *chinensis* management provided they are accurate enough to guide limited surveillance resources to regions with a higher likelihood of *B*. *chinensis* colonization. Dedicating relatively small amounts of surveillance effort to regions that are misidentified as high risk and/or the model failing to identify the occasional high‐risk location are not highly problematic scenarios assuming that surveillance efforts overall are improved by model predictions. Existing studies support the use of SDMs in this way. In particular, while validation of initial SDMs of invasive species is relatively scarce, past work has retrospectively assessed SDM predictions after an expanded invasion and found predictions to be accurate (Barbet‐Massin et al. 2018). In addition, there is an increasing interest in developing iterative SDM methods, wherein early invasion SDMs are updated as the invasion progresses, with early SDMs helping to inform management while management efforts provide data to improve SDMs (Uden et al. 2015; Cook et al. 2019). All this suggests that SDMs can be useful to guide proactive management of predicted suitable habitat before the invasion has a chance to proliferate.

Beyond guiding management and eradication programs, our current work also highlights how developing spatially resolved SDMs in regions of complex topology can be useful for teasing apart the role of environmental variables on species distributions. More specifically, mountain ranges and other landscape features (e.g., large bodies of water that moderate temperature) often disrupt broader scale relationships between environmental factors like temperature and precipitation, temperature and humidity or even temperature and daylight hours. Thus, considering SDMs at high resolution over complex topology provides a relatively underutilized solution for better delineating the environmental variables driving species distributions. In our case, this technique was used to determine the environmental factors limiting an invasive species. However, similar methods could be used, potentially even more easily, for understanding the distributions of native species.

## 5 Conclusions

In summary, our study of *B*. *chinensis* in GSMNP suggests both opportunity and risk. Many regions of the southeastern U.S. are overrun with *B*. *chinensis* . Our analysis indicates that the combined high precipitation and cold winter temperature that characterize much of the southern Appalachians generate natural resistance to *B*. *chinensis* invasion. We see this both in our SDM models and at the Oconaluftee Visitor Center, where *B*. *chinensis* has been established since at least 2001, and yet has only achieved a modest area of invasion. Another promising finding of our study is that the *B*. *chinensis* invasion into GSMNP appears to be in its early stages. Many seemingly suitable regions remain uncolonized, even including some highly disturbed, high‐traffic areas. Thus, there may be enough time to prevent invasion in even the more susceptible low‐elevation regions of the park.

Despite the many optimistic findings of our study, we also find some alarming results. First, a sizeable portion of GSMNP, particularly along the northern border, appears to be susceptible to *B*. *chinensis* invasion. Unfortunately, this is also a high‐traffic region, where opportunity for human translocation is most likely, for example, due to illegal movement of firewood. Second, although the high‐elevation regions of the southern Appalachians appear to provide an *A*. *rudis* *s*.*l*. refuge, the area of this refuge is small (9% of the southeastern U.S.) and represents a tiny fraction of *A*. *rudis* *s*.*l*.'s formerly wide range across the entire East Coast. Third, even if *A*. *rudis* *s*.*l*. can survive, many of its ecosystem services may be lost, especially if most of this region's myrmecochorous plant diversity is not contained within high‐elevation refugia. This would not be the first time that invasive species have been found to disrupt ecosystem‐level processes within and beyond ant systems, and our findings add to a growing body of literature suggesting that interactions between invasive species and keystone natives may result in a greater loss of biodiversity outside of the focal native species due to disruptions of mutualisms and other native community interactions (Warren et al. 2015; Sovie et al. 2016; Fischman, Smyth, and Angelini 2024).

Both in GSMNP, and in the southern Appalachians more broadly, the solution to the *B*. *chinensis* invasion is management. Currently, there is a window of opportunity for eradicating *B*. *chinensis* from the region through a combination of surveillance and removal. Surveillance should focus on locations with high visitation, primarily at low elevations and, based on the findings from this study, with particular emphasis on north‐facing slopes or other locations where drier conditions persist. When supercolonies are found, their spatial extent should be measured, and they should be eliminated. Several methods have been explored for treating infestations, including prey‐baiting with fipronil and hydramethylnon bait (Buczkowski 2016). These methods need to be tested in southern Appalachian field sites, both to assess effectiveness and to determine off‐target impacts on other litter invertebrates. If negative effects can be limited, these methods should be deployed immediately. A combination of favorable climate and early detection means that controlling *B*. *chinensis* within the southern Appalachians is feasible if elimination efforts are put into place quickly. However, if action is not taken soon, we risk missing the opportunity to control the *B*. *chinensis* invasion, potentially enabling the extirpation of valuable ant‐plant mutualisms that are important for structuring understory plant communities in a biodiversity hotspot.

## Author Contributions

**Drew Kanes:** conceptualization (equal), investigation (equal), methodology (equal), writing – review and editing (equal). **Daniel Malagon:** conceptualization (equal), data curation (equal), formal analysis (equal), funding acquisition (equal), investigation (equal), methodology (equal), project administration (equal), writing – original draft (equal), writing – review and editing (equal). **Ben Camper:** conceptualization (equal), data curation (equal), investigation (equal), methodology (equal), project administration (equal). **Anna Hewitt:** investigation (equal), methodology (equal). **Simon Dunn:** conceptualization (equal), investigation (equal), methodology (equal). **Eva Purcell:** investigation (equal), methodology (equal). **Sharon Bewick:** conceptualization (equal), data curation (equal), formal analysis (equal), funding acquisition (equal), investigation (equal), methodology (equal), project administration (equal), resources (equal), writing – original draft (equal), writing – review and editing (equal).

## Conflicts of Interest

The authors declare no conflicts of interest.

## Supporting information


Appendix S1.

Appendix S2.

Appendix S3.

Appendix S4.

Appendix S5.

Appendix S6.

Appendix S7.

Appendix S8.

Appendix S9.

Appendix S10.

Appendix S11.

Appendix S12.

Appendix S13.

Appendix S14.

Appendix S15.

Appendix S16.

Appendix S17.

Appendix S18.

Appendix S19.

Appendix S20.

Appendix S21.

Appendix S22.

## Data Availability

All data, maxent outputs, and r scripts are submitted with this manuscript. Any questions regarding data accessibility can be directed toward the corresponding author.
