# Supplementary figures and images for: Species Distribution Models Reveal Varying Degrees of Refugia From the Invasive Asian Needle Ant for Native Ants Versus Ant‐Plant Seed Dispersal Mutualisms
Source: Ecol Evol. 2025 Jan 16;15(1):e70750. doi: 10.1002/ece3.70750 (PMC11739460; doi:10.1002/ece3.70750)

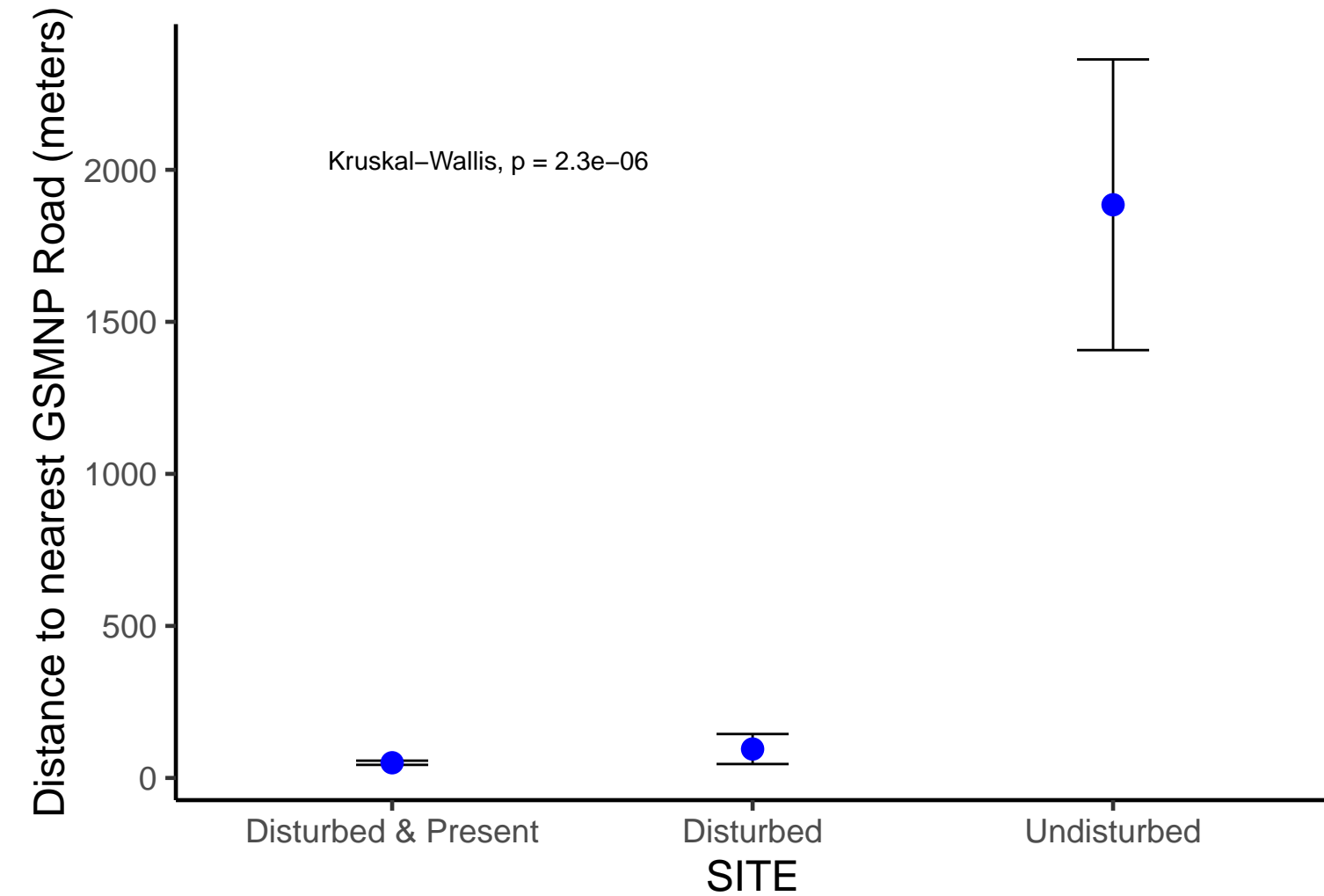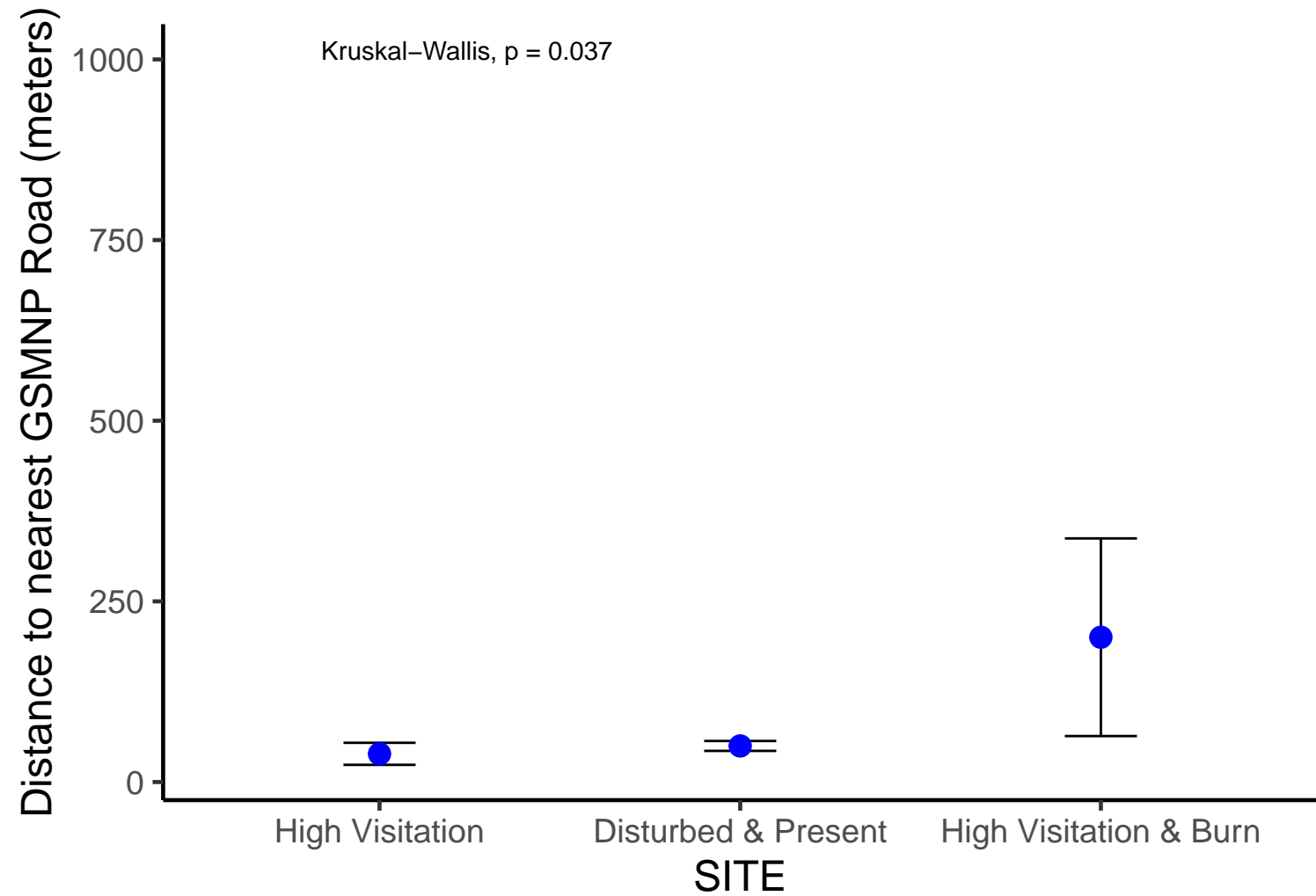

Supplement: Supplementary file 11 — Appendix S11. [file ECE3-15-e70750-s005.pdf]
