# Supplementary material for: Species Distribution Models Reveal Varying Degrees of Refugia From the Invasive Asian Needle Ant for Native Ants Versus Ant‐Plant Seed Dispersal Mutualisms
Source: Ecol Evol. 2025 Jan 16;15(1):e70750. doi: 10.1002/ece3.70750 (PMC11739460; doi:10.1002/ece3.70750)
Supplement: Supplementary file 14 — Appendix S14. [file ECE3-15-e70750-s004.pdf]

Replicated maxent model for aphaenogaster

This page summarizes the results of 25 split-sample models for aphaenogaster, created Wed Dec 15 13:50:27 EST 2021 using Maxent version 3.4.4. The individual models are here: [0] [1] [2] [3] [4] [5] [6] [7] [8] [9] [10] [11] [12] [13] [14] [15] [16] [17] [18] [19] [20] [21] [22] [23] [24]

Pictures of the model

The following two pictures show the point-wise mean and standard deviation of the 25 output grids. Other available summary grids are min, max, median and 95% confidence level (lowerci).

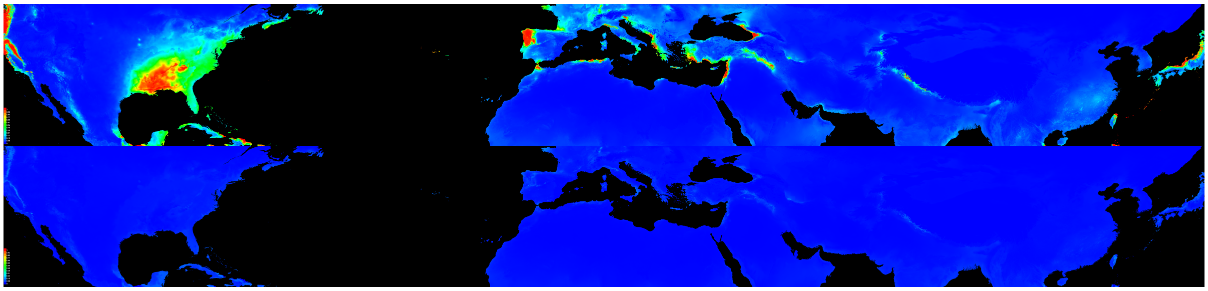

Response curves

These curves show how each environmental variable affects the Maxent prediction. The curves show how the predicted probability of presence changes as each environmental variable is varied, keeping all other environmental variables at their average sample value. Click on a response curve to see a larger version. Note that the curves can be hard to interpret if you have strongly correlated variables, as the model may depend on the correlations in ways that are not evident in the curves. In other words, the curves show the marginal effect of changing exactly one variable, whereas the model may take advantage of sets of variables changing together. The curves show the mean response of the 25 replicate Maxent runs (red) and and the mean +/- one standard deviation (blue, two shades for categorical variables).

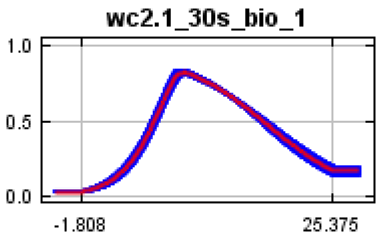

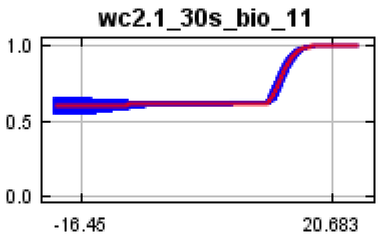

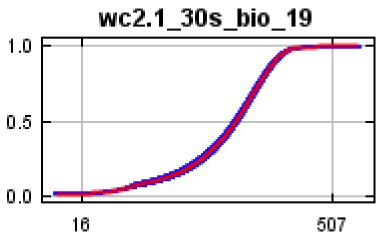

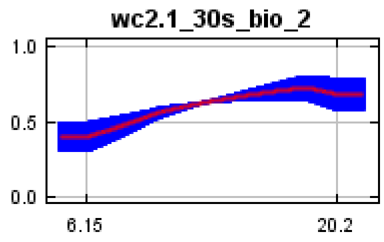

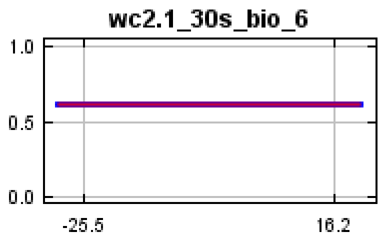

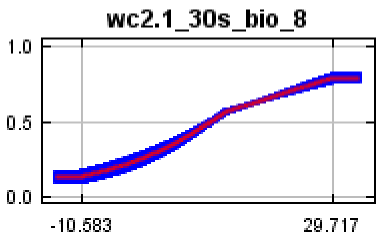

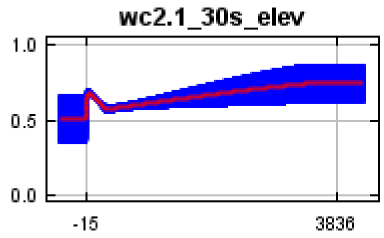

In contrast to the above marginal response curves, each of the following curves represents a different model, namely, a Maxent model created using only the corresponding variable. These plots reflect the dependence of predicted suitability both on the selected variable and on dependencies induced by correlations between the selected variable and other variables. They may be easier to interpret if there are strong correlations between variables.

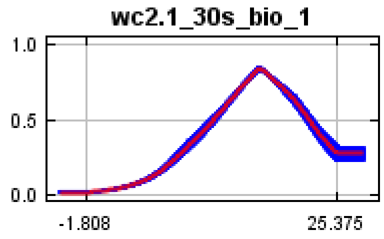

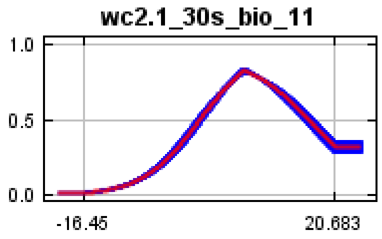

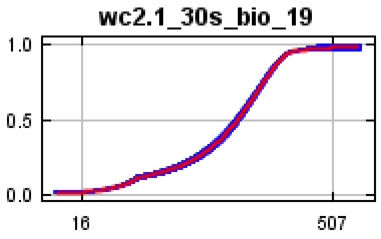

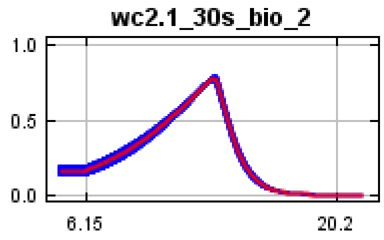

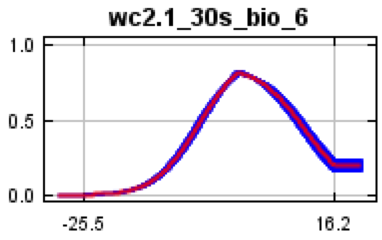

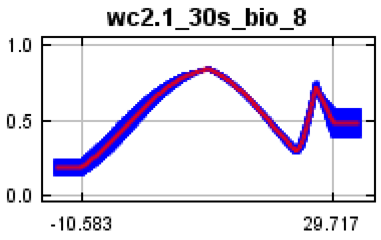

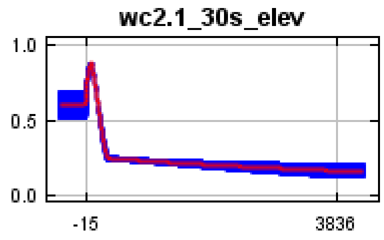

Analysis of variable contributions

The following table gives estimates of relative contributions of the environmental variables to the Maxent model. To determine the first estimate, in each iteration of the training algorithm, the increase in regularized gain

is added to the contribution of the corresponding variable, or subtracted from it if the change to the absolute value of lambda is negative. For the second estimate, for each environmental variable in turn, the values of that variable on training presence and background data are randomly permuted. The model is reevaluated on the permuted data, and the resulting drop in training AUC is shown in the table, normalized to percentages. As with the variable jackknife, variable contributions should be interpreted with caution when the predictor variables are correlated. Values shown are averages over replicate runs.

Variable	Percent contribution	Permutation importance
wc2.1_30s_bio_19	79	82.5
wc2.1_30s_bio_1	10.2	9.9
wc2.1_30s_bio_2	4.2	0.5
wc2.1_30s_bio_11	3.8	2.9
wc2.1_30s_bio_8	1.3	3.5
wc2.1_30s_bio_6	1	0
wc2.1_30s_elev	0.5	0.8

The following picture shows the results of the jackknife test of variable importance. The environmental variable with highest gain when used in isolation is wc2.1_30s_bio_19, which therefore appears to have the most useful information by itself. The environmental variable that decreases the gain the most when it is omitted is wc2.1_30s_bio_19, which therefore appears to have the most information that isn't present in the other variables. Values shown are averages over replicate runs.

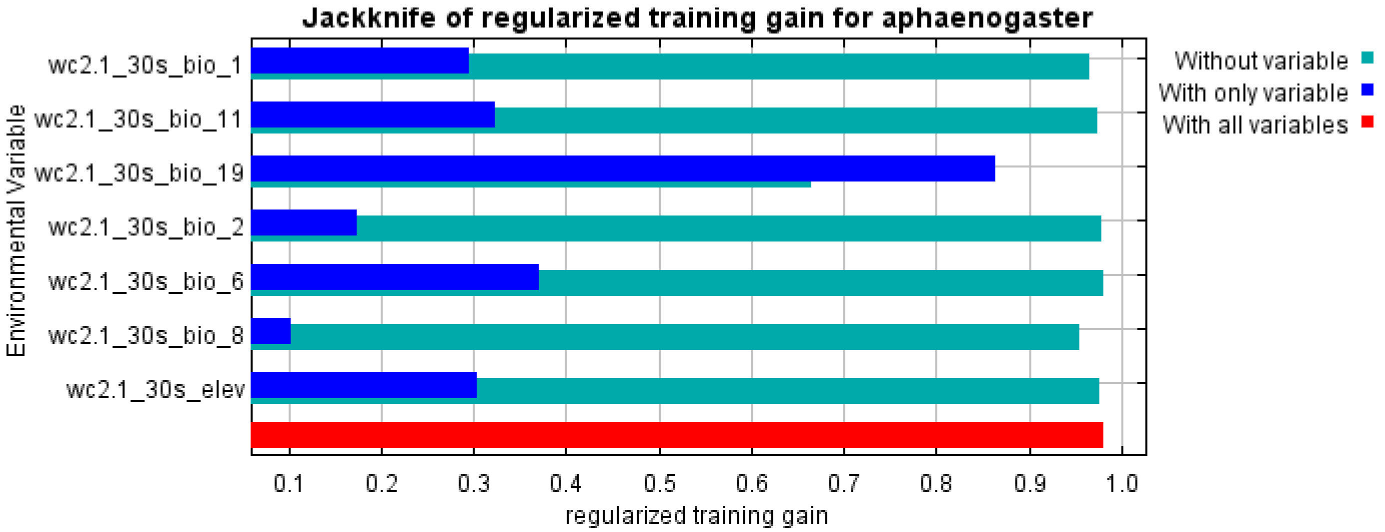

The next picture shows the same jackknife test, using test gain instead of training gain. Note that conclusions about which variables are most important can change, now that we're looking at test data.

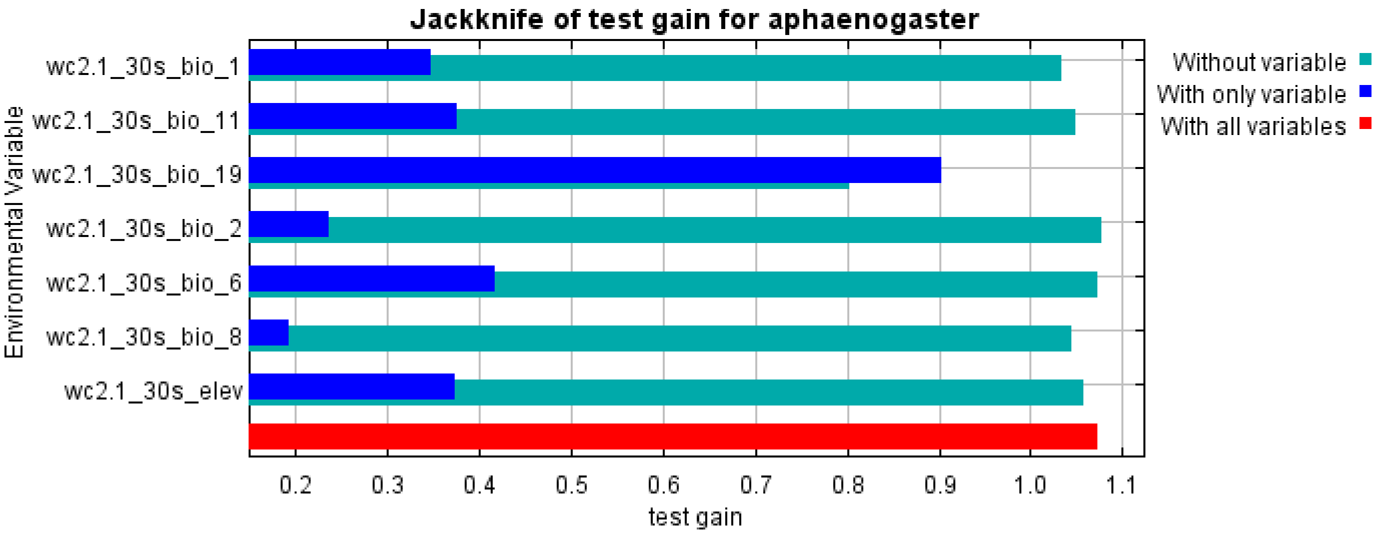

Lastly, we have the same jackknife test, using AUC on test data.

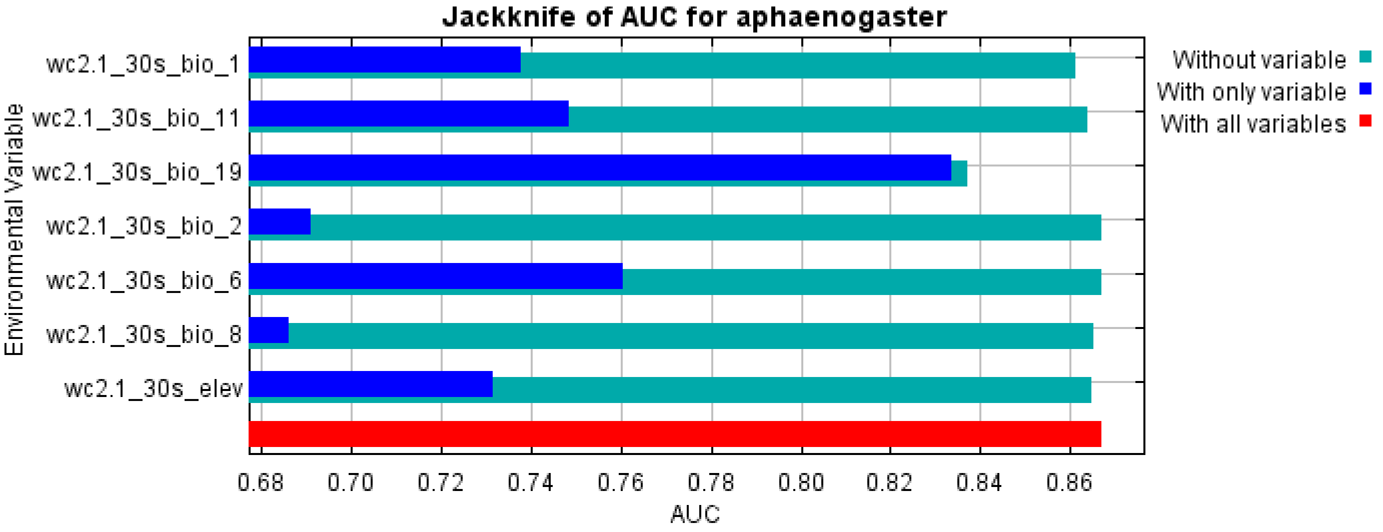

Command line to repeat this species model: java density.MaxEnt nowarnings noprefixes -E "" -E aphaenogaster responsecurves jackknife outputdirectory=C:\Users\anima\OneDrive\Desktop\Aphaenogaster_Maxent_Output samplesfile=C:\Users\anima\OneDrive\Desktop\Rarified_Points\Maxent_SDM_Aphaenogaster.csv environmentallayers=C:\Users\anima\OneDrive\Desktop\Clipped randomseed randomtestpoints=25 betamultiplier=3.0 biasfile=C:\Users\anima\OneDrive\Desktop\Aphaenogaster_Buffer\aphaenogaster.asc replicates=25 replicatetype=subsample nooutputgrids noplots maximumiterations=5000 biastype=3 "applythresholdrule=10 percentile training presence"
