# Supplementary material for: Species Distribution Models Reveal Varying Degrees of Refugia From the Invasive Asian Needle Ant for Native Ants Versus Ant‐Plant Seed Dispersal Mutualisms
Source: Ecol Evol. 2025 Jan 16;15(1):e70750. doi: 10.1002/ece3.70750 (PMC11739460; doi:10.1002/ece3.70750)
Supplement: Supplementary file 19 — Appendix S19. [file ECE3-15-e70750-s018.docx]

SI Figures


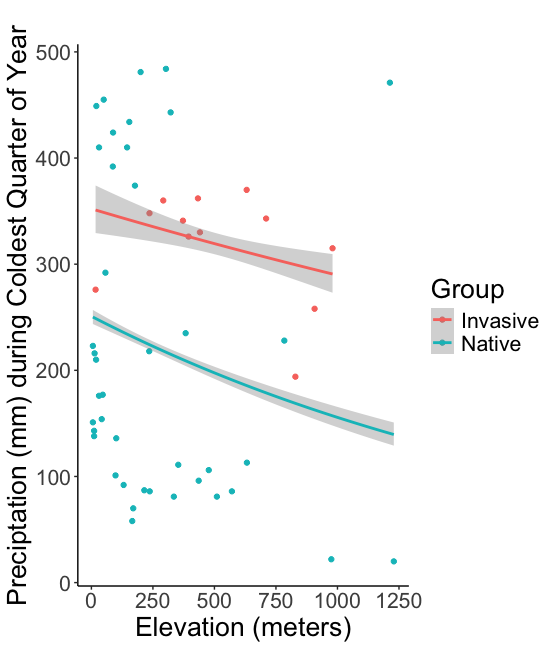


**SI Figure 1.** Differences in precipitation during coldest quarter of the year between needle ant presences in or around Great Smoky Mountains National Park “Invasive” and needle ant presences in southeast Asia “Native.”

**b**

**a**

**SI Figure 2.** A) Mean distance and standard error of the mean between each site type and the nearest GSMNP road. A Kruskal-Wallis test indicated a significant difference in mean distance to a road between 3 site types: Disturbed & Present, Disturbed, and Undisturbed (chi-squared = 25.957, df = 2, p-value = 2.309e-06). Post-hoc Dunn tests with Benjamini-Hochberg p-adjustments revealed significant differences between Disturbed & Present sites and Undisturbed sites (Z= -2.72, adjusted p-value = 9.81e-03) as well as between Disturbed and Undisturbed sites (Z = -5.23 adjusted p-value = 5.07e-07). We found no significant difference in distance to nearest roads between disturbed sites where *B. chinensis* was present and absent (Z= -0.49, adjusted p-value = 6.26e-01). B) A Kruskal Wallis test indicated a significant difference in mean distance to a road between different disturbance types (chi-squared = 6.58, df = 2, p-value = 0.037). Post-hoc Dunn tests with Benjamini-Hochberg p-adjustments revealed marginally significant differences only between High Visitation sites and Burnt & High Visitation sites (Z = -2.31, adjusted p-value = 0.06).
